# Evaluation of an Immersive Virtual Reality Safety Training Used to Teach Pedestrian Skills to Children With Autism Spectrum Disorder

**DOI:** 10.1007/s40617-019-00401-1

**Published:** 2019-12-13

**Authors:** Dennis R. Dixon, Christopher J. Miyake, Karen Nohelty, Marlena N. Novack, Doreen Granpeesheh

**Affiliations:** Center for Autism and Related Disorders, 21600 Oxnard Street, Suite 1800, Woodland Hills, CA 91367 USA

**Keywords:** Autism spectrum disorder, Virtual reality, Safety, Pedestrian, Street crossing

## Abstract

Children with autism spectrum disorder (ASD) are at an increased risk of injury, making safety skills training essential. Whether such training is conducted in the natural environment or in contrived settings is an important consideration for generalization and safety purposes. Immersive virtual reality (VR) environments may offer the advantages of both contrived and natural environment training settings, providing structure to create repeated learning opportunities in a safe and realistic analogue of the natural environment. The current study evaluated the effectiveness of an immersive VR safety skills training environment in teaching 3 children with ASD to identify whether it is safe to cross the street. After modifications to the VR training environment, all 3 participants reached mastery criteria in both VR and natural environment settings. Findings suggest that immersive VR is a promising medium for the delivery of safety skills training to individuals with ASD.

Autism spectrum disorder (ASD) is a pervasive developmental disorder that is characterized by deficits in social communication and social interaction, as well as by restrictive or repetitive patterns of behavior, interests, and activities (American Psychiatric Association, 2013). The deficits characteristic of ASD likely contribute to the increased risk of injury (Lee, Harrington, Chang, & Connors, 2008) and injury-related mortality (Guan & Li, 2017) experienced by diagnosed individuals. As such, it is imperative to incorporate safety skills training into treatment programs for individuals with ASD.

There has been an increase in safety skills training research for individuals with ASD in recent years. In 2010, Dixon, Bergstrom, Smith, and Tarbox conducted a review of safety skills training for individuals with developmental disabilities and identified a number of studies that evaluated behavioral and educational safety skills trainings on emergency situations, accident prevention, and pedestrian skills; however, at the time of that review, very few studies included participants with ASD diagnoses. Since that review, many more studies have focused on safety skills training for individuals with ASD, which have addressed skills for abduction prevention and appropriate response to lures from strangers (Akmanoglu & Tekin-Iftar, 2011; Bergstrom, Najdowski, & Tarbox, 2014; Gunby, Carr, & LeBlanc, 2010; Gunby & Rapp, 2014; Ledbetter-Cho et al., 2016), pedestrian safety (Harriage, Blair, & Miltenberger, 2016; Josman, Ben-Chaim, Friedrich, & Weiss, 2008; Saiano, Garbarino, Lumachi, Solari, & Sanguineti, 2015; Saiano, Garbarino, Lumachi, Solari, & Sanguineti, 2015), lost skills (Bergstrom, Najdowski, & Tarbox, 2012; Carlile, DeBar, Reeve, Reeve, & Meyer, 2018; Hoch, Taylor, & Rodriguez, 2009), first aid (Ergenekon, 2012; Kearney, Brady, Hall, & Honsberger, 2018), household safety (Rossi, Vladescu, Reeve, & Gross, 2017; Summers et al., 2011), water safety (Alaniz, Rosenberg, Beard, & Rosario, 2017; Levy, Ainsleigh, & Hunsinger-Harris, 2017), fire safety (Garcia, Dukes, Brady, Scott, & Wilson, 2016), and general safety and body safety concepts for the prevention of sexual assault (Kenny, Bennett, Dougery, & Steele, 2013). With some exceptions, recent safety skills research has mainly incorporated behavioral techniques, such as instruction, video or live modeling, role-playing, praise, and corrective feedback. The training settings have included homes, classrooms, communities, or a combination of these settings.

An important consideration for safety skills training is whether to conduct training in contrived or natural environment settings (Dixon et al., 2010). Although contrived settings allow for more control to create learning opportunities, the targeted skill must generalize to the natural environment for training to be considered effective. Training does not serve its purpose if the targeted skill fails to generalize. Given that a lack of safety skills may put the individual’s life at risk, the requirement that skills learned in controlled settings must generalize to the natural environment is all the more important. Multiple studies evaluating safety skills training programs conducted in contrived settings (e.g., home, center, classroom) have found additional in situ training to be necessary for skills to generalize for some participants (Bergstrom et al., 2014; Gunby & Rapp, 2014; Himle, Miltenberger, Flessner, & Gatheridge, 2004). For this reason, it may be ideal to conduct training in the natural environment; however, particularly for safety skills training, the natural environment may pose serious challenges as the environment may be dangerous (e.g., crossing a street) or ethically not feasible (e.g., treating a cut, responding to a fire).

Virtual reality (VR) environments may be a compromise between contrived and natural environment settings. VR environments are computer-based, multisensory, simulated environments that are navigated using different technologies (Mesa-Gresa, Gil-Gómez, Lozano-Quilis, & Gil-Gómez, 2018). In recent years, VR has emerged as a promising tool for ASD treatment and has been primarily used to teach social, communication, daily living, and cognitive skills (den Brok & Sterkenburg, 2015; Mesa-Gresa et al., 2018). VR environments may be a way to create an analogue of the natural environment, which may be beneficial for generalization. In addition, VR environments may be easily manipulated to create repeated learning opportunities. Finally, particularly for safety skills training, VR environments pose fewer safety risks than the natural environment.

Street crossing is an important safety skill for individuals with ASD. Given that training on actual streets poses safety concerns, VR may be a practical training medium for this skill. Preliminary research has been conducted on the use of VR to teach pedestrian skills to individuals with ASD. Strickland, McAllister, Coles, and Osborne (2007) described a VR environment used to teach street safety skills to two children with ASD. Participants navigated the VR environment via physical actions (e.g., walking, stopping, turning head) using a head-mounted display, body tracker, and three-dimensional hand controls; however, the cables that connected the headset to the computer were found to pose a safety concern. The VR environment displayed a street, stop sign, and moving cars. Training, which involved physical prompting to help participants stop at the stop sign and track moving cars, was conducted over approximately twenty 3- to 5-min sessions over a 5-week period. Although the participants learned new behaviors (e.g., tracking cars, locating the stop sign) in the immersive VR environment, generalization to the natural environment was not tested. Josman et al. (2008) evaluated the effectiveness of a desktop VR environment for teaching street-crossing skills to six children with ASD and a control group of six children with typical development. The VR street environment displayed an avatar facing the crosswalk of a four-lane street with cars traveling left and right. The scene was navigated using three keyboard keys to orient left, orient right, and move forward across the street. Participants practiced crossing the virtual street and progressed at their own pace through the VR software, which incorporated nine stages that increased in difficulty (i.e., a greater number of cars traveling at faster speeds). Participants interacted with the VR environment for approximately eight 10- to 30-min sessions. The participants with ASD were found to demonstrate improvements in street-crossing skills in the VR environment, and half of the participants with ASD showed improvement in a protected street-crossing setting as compared to baseline data.

More recently, Saiano, Garbarino, et al., (2015) compared two different interfaces for their VR street-crossing environment. Participants in these studies were adults with ASD who interacted with the same VR environment using either gestural (i.e., Microsoft Kinect) or game pad interfaces. The VR environment was displayed in first-person perspective and depicted a city with buildings, sidewalks, streets, traffic lights, crosswalks, road signs, and cars that were static in the scene. Participants were asked to navigate to a specific location (e.g., the police station) in the VR environment by following signs and using crosswalks. Training was conducted over ten 45-min sessions. Although performance within the VR environment was found to improve with both interfaces, the game pad interface was determined to be easier for participants to use based on average navigation speeds. Posttreatment questionnaires, however, showed no significant improvement for participants using either interface. Parent questionnaires were used to assess generalization to the natural environment. Based on parent report, the gesture-based interface was found to be superior to the game pad interface, though no direct testing of generalization was conducted in either study. A limitation of the available research on VR street-crossing training is a lack of testing in the natural environment. When tested (Josman et al., 2008), generalization was demonstrated by only half of the participants with ASD, which is not ideal for such an important safety skill. One explanation for the limited effectiveness observed across these studies may be related to the level of immersion used.

Level of immersion differs across various VR interfaces. In a recent review of research on the use of VR to teach social skills to individuals with ASD, Miller and Bugnariu (2016) defined different levels of VR immersion (i.e., low, moderate, and high) based on a number of criteria, including the number of signals indicating the presence of devices, the number of senses involved, the type of interface, visual resolution, and motion-capturing capability. VR immersion was classified as high if it had limited signals indicating the presence of devices, involved more than two senses, incorporated a head-mounted device or surround projection, had a high visual resolution, and involved full-body motion capture. Although very little high-immersion VR research was found, the existing research did reveal a strong treatment response. High-immersion VR may have benefits over low-immersion VR for teaching complex skills, such as those that require gestures (Miller & Bugnariu, 2016). For street-crossing training, immersive VR incorporating a head-mounted device, for example, may have greater advantages than VR displayed on a computer monitor. With a head-mounted display, the target behaviors more closely match the natural environment (i.e., actually turning one’s head to look left and right to see if a car is coming), as opposed to a computer screen where the actual physical behaviors are not practiced (i.e., one’s head remains fixed toward a screen). In this case, the head-mounted display produces a more accurate analogue of the natural environment.

Although there has been an increase in safety skills training research for individuals with ASD in the last decade, more research is needed on different skills and mediums of training. The purpose of the current study is to evaluate the effectiveness of an immersive VR training environment in teaching children with ASD to identify whether it is safe to cross a street in the natural environment.

## Method

### Participants

All participants were receiving applied behavior analysis services from the same center-based clinic in California. During the course of the study, pedestrian skills were not concurrently targeted during ongoing treatment for any of the participants. Potential participants were considered for inclusion in the study if both the child’s clinical supervisor and caregivers agreed that street-crossing skills were appropriate to target. To be included in the current study, participants must have met the following criteria: (a) had a diagnosis of ASD (American Psychiatric Association, 2013) by a licensed professional (e.g., licensed psychologist, pediatrician, neurologist); (b) used spoken language as his or her primary mode of communication; (c) was receiving primarily center-based services, with at least three scheduled sessions per week; (d) had prerequisite skills including following instructions, basic compliance, and maintaining attention; (e) had no parent-reported visual or auditory impairments; (f) had English as his or her primary household language; (g) tolerated the head-mounted device following a desensitization protocol; and (h) demonstrated lack of street-crossing skills during baseline probes. One child was excluded from the study because he did not tolerate the head-mounted device following the desensitization protocol.

Three children participated in the current study. Joe was a 4-year-old male diagnosed with ASD. He spoke in five- to eight-word full sentences to mand and tact about his environment. Joe was able to answer yes/no questions and simple *who*, *what*, and *where* questions. Kaiden was a 6-year-old male with ASD. He typically engaged in three- to five-word sentences and phrasal speech to mand and tact. Kaiden was able to answer yes/no questions and follow one-step instructions. Bob was a 10-year-old male diagnosed with ASD and attention deficit hyperactivity disorder. He typically spoke in three- to four-word phrases to mand. Bob was able to answer simple yes/no questions. See Table 1 for a summary of participant characteristics and Pervasive Developmental Disorder Behavior Inventory (PDDBI; Cohen, Schmidt-Lackner, Romanczyk, & Sudhalter, 2003) scores. The PDDBI is an assessment designed to measure problem behaviors and social communication skills associated with ASD. When interpreting PDDBI scores, it is important to note that the PDDBI was standardized on children with ASD; thus, a mean score of 50 is representative of children with ASD (Cohen & Sudhalter, 2005). For the composite score and subscales measuring problem behaviors, higher scores indicate greater levels of severity. For the subscales measuring skills, higher scores indicate greater levels of ability. Joe’s profile on the PDDBI indicated strengths in the areas of expressive language and learning, memory, and receptive language. His level of ASD severity is consistent with high-functioning ASD. Kaiden and Bob’s composite and subscale scores all fell within the average range for children with ASD.

**Table 1:** Participant Information

Participant	Age (Years)	Diagnosis	PDDBI Scores				
			SENSORY	SEMPP	EXPRESS	LMRL	Autism Composite
Joe	4	ASD	42	41	71	62	27
Kaiden	6	ASD	50	49	58	59	43
Bob	10	ASD & ADHD	48	58	52	51	52

*Note.* ASD = autism spectrum disorder; ADHD = attention deficit hyperactivity disorder; PDDBI = Pervasive Developmental Disorder Behavior Inventory; SENSORY = sensory/perceptual approach behaviors; SEMPP = semantic/pragmatic problems; EXPRESS = expressive language; LMRL = learning, memory, and receptive language.

### Settings and Materials

Baseline and natural environment sessions were conducted in the natural environment. Sessions took place outside on streets in the community. The streets were located in either the participants’ neighborhoods or near the center (i.e., not random locations). All three participants’ neighborhoods were in suburban housing developments. The residential streets for two of the three participants did not have sufficient traffic to conduct trials, so baseline and natural environment sessions were performed at a nearby park or school. Between two and three different streets were used for each participant across baseline and natural environment conditions. Sessions were conducted at various times of the day during daylight hours (i.e., after sunrise and before sunset). No modifications to the natural environment were made, with the exception of having a research assistant drive a car on the street if no other cars were present.

Training sessions were conducted in the clinic in a clinic room that measured 10 ft. by 12 ft. (3.05 m by 3.66 m) and was equipped with a table and an Oculus Rift (Version CV1; 2016) headset and sensors. The Oculus Rift was connected to a laptop, which was used by the clinician to view what the participant was seeing in the head-mounted device for scoring and training purposes. The sensors were also used by the clinician to set up training trials.

The immersive VR environment consisted of 360-degree videos of real streets from the participants’ community. Videos were recorded using a Samsung Gear 360 (2017) camera attached to a 4-ft. (1.2 m) monopod that was placed on the edge of the sidewalk about 2 to 3 ft. (0.6 to 0.9 m) from the street. A low-profile monopod was used to reduce visual distractions in the videos. The videos were captured in 5- to 10-min segments and then edited down to the desired clip length using a video editing program. Multiple exemplars of streets were incorporated, with eight different streets used for the short video clips and two different streets used for the long video clips. The videos were hosted on YouTube (https://www.youtube.com/) and accessed through Oculus Rift (Version 1.3; 2016) and Steam Client (2018) software.

### Data Collection and Interobserver Agreement

During VR training conditions, participants were evaluated on their performance of completing three steps. The first step was to look left and right. A correct response was defined as moving the head to the left and to the right at least 45 degrees from center within 5 s of the presentation of the street in the immersive VR environment. The second step was responding to the question “Is there a car moving?” A correct response was defined as responding “yes” when a car was visibly moving anywhere within view or responding “no” when no cars were visibly moving. The third step was responding to the question “Is it safe to cross?” A correct response was defined as responding “yes” if no cars were visibly moving or responding “no” if any cars were visibly moving anywhere within view. For training conditions, data were collected on all three steps to provide instruction on salient features. During baseline, natural environment probe, and VR probe conditions, participants were evaluated on only the third step (i.e., response to the question “Is it safe to cross?”) to assess the use of the safety skill under more natural conditions (e.g., a caregiver is not likely to go through the three training steps with a child before crossing the street).

Data were collected using a paper data sheet. Participants’ responses for each applicable step were scored as correct, incorrect, or prompted. For each session, data were summarized by calculating the percentage correct across applicable steps. Although the targeted skill was to discriminate between safe and unsafe conditions, and not to safely cross the street, a misjudgment in the natural environment could potentially have severe consequences (e.g., in the event of wandering or eloping). Thus, the mastery criterion was defined as 100% correct across two consecutive sessions for VR training, one session for VR probes, and three consecutive sessions for baseline and natural environment probes.

Interobserver agreement (IOA) data were collected for 33% of sessions. Two observers scored each IOA trial as correct, incorrect, or prompted. IOA was calculated by dividing the number of agreements by the total number of agreements plus disagreements and multiplying by 100. An average of 96% IOA per trial coded was found across all conditions, specifically 100% for baseline and natural environment probes (range 100%–100%), 93% for VR probes (range 80%–100%), 100% for VR training sessions (range 100%–100%), and 92% for VR training with long videos (range 78%–100%). Depending on the condition, data were collected on either one response (baseline, natural environment, and VR probes) or three responses (VR training conditions). For VR training conditions, each of the three responses was considered a trial. Across VR training conditions, there was 92% agreement for looking left and right, 96% agreement for identifying a moving car, and 98% agreement for identifying if it was safe or unsafe to cross.

### Procedure

A nonconcurrent multiple-baseline design across participants was used to assess the effects of an immersive VR training environment on the participants’ ability to identify whether a street is safe to cross in the natural environment. The study was conducted in multiple phases; each phase is discussed in turn.

#### Preexperiment desensitization to the VR device

Before the study was initiated, all three participants were introduced to the head-mounted device via desensitization sessions. Participants were given time to become familiar with the head-mounted device via play. Participants were presented with different preferred videos and games to interact with during the desensitization sessions. One participant was known to display defensiveness (i.e., ducking, evading) toward attempts to put clothing or accessories on his head (e.g., hats, glasses). For this participant, a smaller head-mounted device was introduced prior to the Oculus Rift. Participants were considered ready to begin the study once they independently requested to use the Oculus Rift. Desensitization sessions lasted approximately 3 to 5 min. Between two and three desensitization sessions conducted in a single day were required for the participants.

#### Baseline and natural environment probes

Baseline and natural environment probe sessions were conducted on streets in the natural environment. During each trial, the participant and clinician approached the street as if to cross and the clinician presented the probe “Is it safe to cross?” All consequences, including praise and corrective feedback, were withheld, and no prompts were provided. To assure safety, the clinician held the hand or arm of the participant to inhibit the participant from crossing the street. Baseline and natural environment probe sessions consisted of an average of 5.2 trials (range 5–6). Sessions were conducted over the course of 1 to 2 days. A minimum of 5 min elapsed between each session.

#### VR probes

VR probe sessions were conducted in the immersive VR environment. For each trial, the clinician played a video clip. Depending on the training phase, different videos were used for VR probe sessions (see the subsequent sections on the VR training phases for descriptions of the videos used). During the video clip, the clinician presented the probe “Is it safe to cross?” For each trial, all consequences, including praise and corrective feedback, were withheld and no prompts were provided. Each VR probe session consisted of five trials.

#### VR training

VR training sessions were conducted in the immersive VR training environment. For each trial, the clinician played a video clip. Video clips were approximately 10 s in length and showed either a safe or an unsafe situation. Safe situation clips showed no cars passing, whereas unsafe situation clips displayed one or more cars passing. Other than cars parked on the street in some of the videos, no distractions were present in the videos.

Flexible prompt fading was used to teach the participants the approach responses for all three steps (i.e., looking left and right, responding to “Is there a car moving?”, and responding to “Is it safe to cross?”; Leaf, Cihon, Leaf, McEachin, & Taubman, 2016). Flexible prompt fading involves the clinician using his or her judgment in selecting a prompt, if any, to provide for a given trial. General guidelines (e.g., using the least intrusive prompt, providing prompts to support the individual in responding correctly on at least 80% of trials) are followed in lieu of a prespecified prompting sequence. This method was chosen so that the researchers could use their clinical judgment in the moment to individualize prompts for each participant. Because VR is a new technology with unknown challenges, a more dynamic approach was chosen over a static prompting hierarchy. Regardless of a correct or a prompted response, verbal praise was provided after each step, verbal praise or a token was provided at the end of each trial, and access to a preferred activity (e.g., games or videos on the headset) was provided after each session based on the participant’s preference.

A training session lasted between 3 and 5 min, during which time an average of 5.46 trials (range 3–8) was presented; in addition to the presentation of trials (i.e., video clips), the clinician conducted a preference assessment, gained the participant’s attention before each trial, and provided reinforcement or corrective feedback after each response. Training sessions were conducted at least 1 hr apart, and up to four sessions were conducted in a single day. Training occurred over 2 to 3 days in a 1-week period.

As the study continued, adaptations were made to the training procedures to aid generalization to the natural environment. Two additional training phases were added.

- ***VR training with distractors*.** During the VR training with distractors phase, the original video clips were modified. It was hypothesized that noises present in the natural environment were causing distractions and limiting generalization. Thus, additional distraction audio was laid over the video clips; this audio included sounds of dogs barking, lawn mowers, leaf blowers, and garbage trucks. During the VR training with distractors phase, the training procedures remained the same.
- ***VR training with long videos.*** For the VR training with long videos phase, new videos of streets were recorded. It was hypothesized that the researcher’s behavior of queueing each video was serving as the discriminative stimulus (S^D^) in the previous training trials, suggesting that the probe (i.e., “Is it safe to cross?”) in the presence of the street did not have appropriate stimulus control. To more accurately represent the natural environment and aid generalization, 4- to 5-min video clips were recorded, which also included overlaid distraction audio (e.g., dogs barking, lawn mowers, leaf blowers, and garbage trucks). Rather than presenting a single trial per video clip as previously described, the long videos were used to probe an average of 5.6 trials (range 3–8). Only one long video was played per training session. In addition, slightly different training procedures were used. As the video played, each trial was initiated by the clinician asking, “Is it safe to cross?” Flexible prompt fading was then used to guide the participant through all three steps.

## Results

**Fig. 1:**
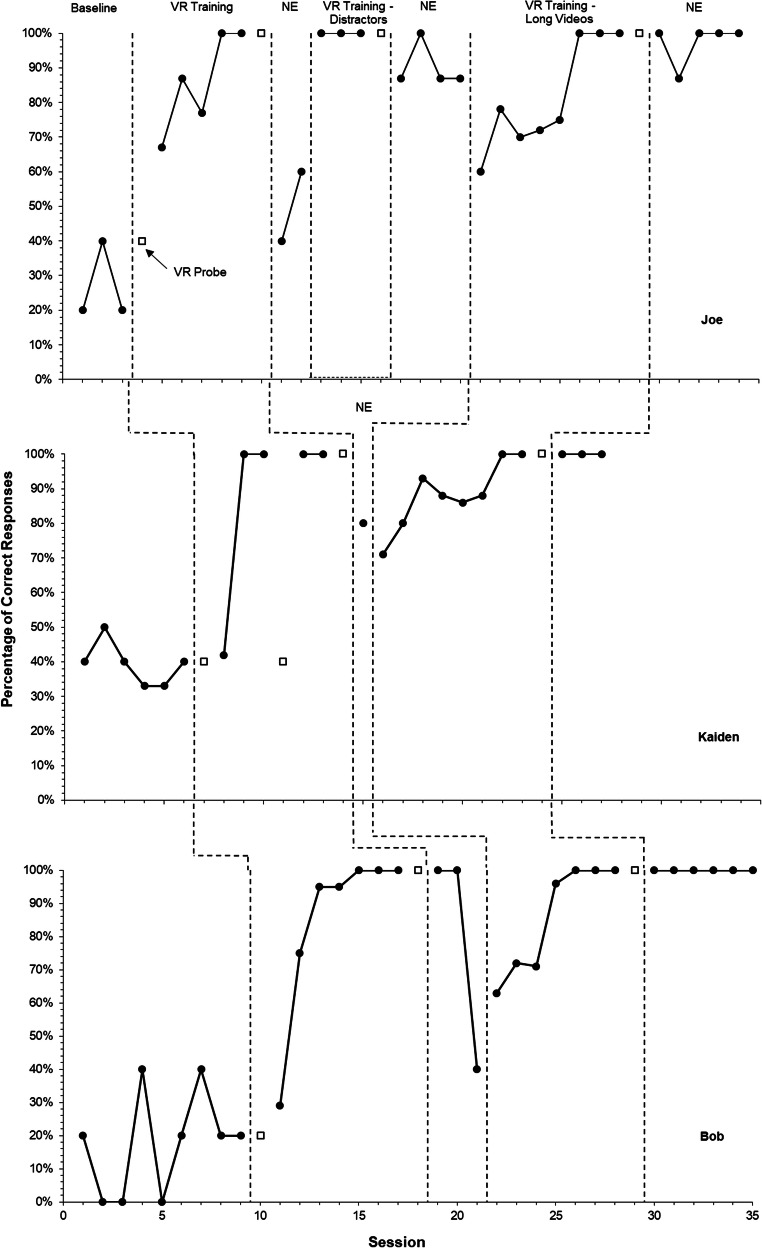
Percentage of correct responses during baseline, natural environment (NE), and virtual reality (VR) probe and training conditions

A nonconcurrent multiple-baseline design was used to assess the effectiveness of VR training. Table 2 presents a detailed summary of sessions and trials for each participant, and Fig. 1 depicts the results for each participant across baseline, natural environment probes, VR probes, and training conditions.

**Table 2:** Summary of Sessions and Trials for Each Participant

Condition	Joe	Kaiden	Bob
Baseline and natural environment probes			
Total sessions	14	10	15
Total trials	61	56	90
Total safe trials	37	34	61
Total unsafe trials	39	22	29
Percentage of trials correctly identified as unsafe during baseline	0%	58%	70%
VR training conditions			
Total sessions	16	13	14
Total trials	79	79	77
Total safe trials	39	38	38
Total unsafe trials	40	41	39
Initial VR training sessions	5	5	7
Initial VR training trials	25	27	39
VR training with distractors sessions	3	0	0
VR training with distractors trials	15	0	0
VR training with long videos sessions	8	8	7
VR training with long videos trials	39	52	38

During baseline and the initial VR probe, all participants demonstrated low, stable responding, with scores between 0% and 50%. All three participants met the criterion in the initial VR training condition in five, three, and six sessions, respectively, for Joe, Kaiden, and Bob. Joe and Bob immediately met the criterion in the VR probe condition; although Kaiden did not meet the criterion on the subsequent VR probe, he did so after two more sessions in the initial VR training condition. None of the participants demonstrated generalization to the natural environment after the initial VR training condition. Joe was the only participant exposed to the VR training with distractors condition; after meeting the training and VR probe mastery criterion, he again did not demonstrate generalization to the natural environment. Subsequently, Joe, Kaiden, and Bob were exposed to the VR training with long videos condition and met the training criterion in seven, eight, and six sessions, respectively; in the following VR probe, each participant met the criterion. All three participants met the criterion in the natural environment.

Experimental control was demonstrated using a nonconcurrent multiple-baseline design. For each participant in the study, low, stable scores were observed during baseline and an increase in scores was seen after each VR training condition. Unlike Joe, Kaiden and Bob did not show an increase over baseline during their first VR training session. Kaiden and Bob required more prompting in comparison to Joe, which resulted in scores that were similar to baseline levels. Nevertheless, ultimate mastery in the natural environment was displayed for each participant only after meeting the criterion in the VR training with long videos condition. Given the stable baselines and the replication of behavior change across participants, a functional relationship was demonstrated between mastery in the natural environment and the presentation of the sequence of VR training conditions (i.e., three training conditions for Joe and two training conditions for Kaiden and Bob).

## Discussion

The purpose of the current study was to evaluate the effectiveness of an immersive VR training protocol for teaching children with ASD to identify if it is safe to cross the street in the natural environment. Modifications were made to the original training environment, and ultimately all three participants achieved the mastery criterion in the natural environment.

Like previous VR street-crossing research that found participant performance within the VR environment to improve following training (Josman et al., 2008; Saiano, Garbarino, et al., 2015; Strickland et al., 2007), the current study found that participants demonstrated mastery criteria during the VR probes following each of the training conditions. Generalization to the natural environment was also achieved following the VR training with long videos condition. As demonstrated in previous safety skills research, supplementary in situ training has often been required for generalization to occur (Bergstrom et al., 2014; Gunby & Rapp, 2014; Himle et al., 2004). It is noteworthy that in the present study, generalization to the natural environment was achieved with training conducted solely in the immersive VR environment. This is important because a limitation of existing VR street-crossing research and VR-based ASD treatment research overall is that generalization to the natural environment is not often tested (den Brok & Sterkenburg, 2015). Josman et al. (2008) did assess generalization in their evaluation of a desktop VR street-crossing training, which showed improvements for half of the participants with ASD. In the current study, all participants achieved the mastery criterion in the natural environment, which may be attributed to the greater level of immersion used in the training (i.e., head-mounted devices vs. a computer monitor display), as well as to the modifications made to the VR training videos to create a more realistic analogue of the natural environment.

The immersive VR environment used in the current study underwent some modifications before it was found to be effective. Initially, short, 10-s video clips were used in the immersive VR environment to maximize the number of learning trials presented during training sessions. The shorter videos were found to produce mastery in the VR environment, which was consistent across participants; however, these procedures alone failed to fully generalize the skills to the natural environment. During Joe’s natural environment probe session, he was observed orienting toward the direction of various sounds (e.g., dogs barking) during and in between trials. For Joe, it was speculated that generalization did not take place due to the noises and distractions present in the natural environment. Thus, distraction audio was added to the original 10-s video clips. During the VR training with distractions condition, Joe again quickly acquired the skill in the VR environment; however, the skill again failed to generalize to the natural environment.

It was speculated that the abrupt start of each short video clip, rather than the presence of the street, served as the S^D^ for each trial. Thus, longer, 4- to 5-min videos with distraction audio were created to more accurately represent the natural environment. When the video length was extended and multiple trials were presented in a single video, the S^D^ more closely replicated the natural environment. Between trials in the long-videos training condition (i.e., when the video was playing but the researcher was not actively asking questions), all participants were found to lose some focus and divert their attention toward other stimuli in the VR environment (e.g., turn around, look directly up or down). The researcher then prompted the participant for the first response of looking left and right. Although the long videos were found to be more distracting for participants and learning occurred at a slower rate than in the previous VR training phases, the participants were training in an environment that more closely resembled the natural environment, and generalization to the natural environment was ultimately achieved. The authors can only speculate that generalization was attributed to the long-video training condition and not some other element of training (e.g., total intervention time). The long-video format should be implemented on its own in future investigations to see if it contributes to more rapid generalization to the natural environment.

Although the immersive VR technology was found to be effective in the current study, it was not without limitations. Head-mounted devices may not be tolerated by all individuals with ASD. One participant was excluded from the study because he could not complete the desensitization phase. That said, a strength of the current study is the young age of the participants. Research on VR treatment for individuals with ASD has mainly focused on children ages 8 to 14 years old (Mesa-Gresa et al., 2018). The current study demonstrated that, with desensitization procedures, children as young as 4 years old can effectively use head-mounted VR devices for treatment. A further limitation of the technology used in this study is that only the skill of identifying whether a street is safe to cross was trained and evaluated. Additional skills (e.g., actually crossing the street) could not be included without significant software development. Although this is a limitation, it is worth mentioning that the VR technology employed in the current study was fairly easy to use, required essentially no software development, and was reasonably cost effective, which would allow for relatively simple replication and implementation within other clinical settings. Finally, there are general concerns regarding the lack of research and unknown safety with respect to the use of VR head-mounted devices with children (Gent, 2016). These concerns underline the need for supervised usage and limited access to such technology until larger studies demonstrate its safety.

The current study identified a practical immersive VR training environment that was effective in teaching children with ASD to identify whether it is safe to cross a street in the natural environment. One of the strengths of the immersive VR training environment was the ability to target the physical aspect of looking left and right, which cannot be achieved using a computer monitor display (e.g., Josman et al., 2008). Practitioners may leverage this technology to target other skills in which there is an associated motor movement (e.g., scanning an environment for a salient object). This technology may also be useful for teaching other skills that are dangerous to target in the natural environment. For instance, this technology may be used to build upon existing desktop fire-safety trainings (e.g., Padgett, Strickland, & Coles, 2006) that may benefit from an immersive environment to scan for hazards and identify emergency exits. Immersive VR training videos give practitioners control over the entire visual field of the individual, where they can isolate what is most salient, present scenarios that are rare or difficult to contrive in the natural environment, and limit or eliminate distractions; however, when creating VR training videos, practitioners should take into account potential barriers to generalization. This technology offers a good deal of control to contrive learning opportunities; however, overengineering the scenarios may limit generalization to the natural environment. To successfully transition from the VR training environment to the natural environment, without supplemental in vivo training, it may be a good process to work backward from the closest representation of the target environment. Nevertheless, this is a promising technology to teach skills that pose a risk or challenge to target within the natural environment, and further research is warranted.

## References

[CR1] Akmanoglu N, Tekin-Iftar E (2011). Teaching children with autism how to respond to the lures of strangers. Autism.

[CR2] Alaniz ML, Rosenberg SS, Beard NR, Rosario ER (2017). The effectiveness of aquatic group therapy for improving water safety and social interactions in children with autism spectrum disorder: A pilot program. Journal of Autism and Developmental Disorders.

[CR3] American Psychiatric Association (2013). *Diagnostic and statistical manual of mental disorders*.

[CR4] Bergstrom R, Najdowski AC, Tarbox J (2012). Teaching children with autism to seek help when lost in public. Journal of Applied Behavior Analysis.

[CR5] Bergstrom R, Najdowski AC, Tarbox J (2014). A systematic replication of teaching children with autism to respond appropriately to lures from strangers. Journal of Applied Behavior Analysis.

[CR6] Carlile KA, DeBar RM, Reeve SA, Reeve KF, Meyer LS (2018). Teaching help-seeking when lost to individuals with autism spectrum disorder. Journal of Applied Behavior Analysis.

[CR7] Cohen IL, Schmidt-Lackner S, Romanczyk R, Sudhalter V (2003). The PDD Behavior Inventory: A rating scale for assessing response to intervention in children with pervasive developmental disorder. Journal of Autism and Developmental Disorders.

[CR8] Cohen IL, Sudhalter V (2005). *PDD Behavior Inventory: Professional manual*.

[CR9] den Brok WLJE, Sterkenburg PS (2015). Self-controlled technologies to support skill attainment in persons with an autism spectrum disorder and/or an intellectual disability: A systematic literature review. Disability and Rehabilitation: Assistive Technology.

[CR10] Dixon DR, Bergstrom R, Smith MN, Tarbox J (2010). A review of research on procedures for teaching safety skills to persons with developmental disabilities. Research in Developmental Disabilities.

[CR11] Ergenekon Y (2012). Teaching basic first-aid skills against home accidents to children with autism through video modeling. Educational Sciences: Theory and Practice.

[CR12] Garcia D, Dukes C, Brady MP, Scott J, Wilson CL (2016). Using modeling and rehearsal to teach fire safety to children with autism. Journal of Applied Behavior Analysis.

[CR13] Gear 360 [Camera]. (2017). Suwon, South Korea: Samsung Electronics.

[CR14] Gent, E. (2016, October). Are virtual reality headsets safe for children? A lack of data and guidelines is leaving consumers in the dark about virtual reality’s potential negative side effects for kids. *Scientific American.* Retrieved from https://www.scientificamerican.com/article/are-virtual-reality-headsets-safe-for-children/

[CR15] Guan J, Li G (2017). Injury mortality in individuals with autism. American Journal of Public Health.

[CR16] Gunby KV, Carr JE, LeBlanc LA (2010). Teaching abduction-prevention skills to children with autism. Journal of Applied Behavior Analysis.

[CR17] Gunby KV, Rapp JT (2014). The use of behavioral skills training and in situ feedback to protect children with autism from abduction lures. Journal of Applied Behavior Analysis.

[CR18] Harriage B, Blair KSC, Miltenberger R (2016). An evaluation of a parent implemented in situ pedestrian safety skills intervention for individuals with autism. Journal of Autism and Developmental Disorders.

[CR19] Himle MB, Miltenberger RG, Flessner C, Gatheridge B (2004). Teaching safety skills to children to prevent gun play. Journal of Applied Behavior Analysis.

[CR20] Hoch H, Taylor BA, Rodriguez A (2009). Teaching teenagers with autism to answer cell phones and seek assistance when lost. Behavior Analysis in Practice.

[CR21] Josman N, Ben-Chaim HM, Friedrich S, Weiss PL (2008). Effectiveness of virtual reality for teaching street-crossing skills to children and adolescents with autism. International Journal on Disability and Human Development.

[CR22] Kearney KB, Brady MP, Hall K, Honsberger T (2018). Using peer-mediated literacy-based behavioral interventions to increase first aid safety skills in students with developmental disabilities. Behavior Modification.

[CR23] Kenny MC, Bennett KD, Dougery J, Steele F (2013). Teaching general safety and body safety training skills to a Latino preschool male with autism. Journal of Child and Family Studies.

[CR24] Leaf JB, Cihon JH, Leaf R, McEachin J, Taubman M (2016). A progressive approach to discrete trial teaching: Some current guidelines. International Electronic Journal of Elementary Education.

[CR25] Ledbetter-Cho K, Lang R, Davenport K, Moore M, Lee A, O’Reilly M (2016). Behavioral skills training to improve the abduction-prevention skills of children with autism. Behavior Analysis in Practice.

[CR26] Lee LC, Harrington RA, Chang JJ, Connors SL (2008). Increased risk of injury in children with developmental disabilities. Research in Developmental Disabilities.

[CR27] Levy KM, Ainsleigh SA, Hunsinger-Harris ML (2017). Let’s go under! Teaching water safety skills using a behavioral treatment package. Education and Training in Autism and Developmental Disabilities.

[CR28] Mesa-Gresa P, Gil-Gómez H, Lozano-Quilis JA, Gil-Gómez JA (2018). Effectiveness of virtual reality for children and adolescents with autism spectrum disorder: An evidence-based systematic review. Sensors.

[CR29] Miller HL, Bugnariu NL (2016). Level of immersion in virtual environments impacts the ability to assess and teach social skills in autism spectrum disorder. Cyberpsychology, Behavior, and Social Networking.

[CR30] Oculus Rift (Version 1.3) [Computer software]. (2016). Menlo Park, CA: Facebook Technologies.

[CR31] Oculus Rift (Version CV1) [Apparatus]. (2016). Menlo Park, CA: Facebook Technologies.

[CR32] Padgett LS, Strickland D, Coles CD (2006). Case study: Using a virtual reality computer game to teach fire safety skills to children diagnosed with fetal alcohol syndrome. Journal of Pediatric Psychology.

[CR33] Rossi MR, Vladescu JC, Reeve KF, Gross AC (2017). Teaching safety responding to children with autism spectrum disorder. Education and Treatment of Children.

[CR34] Saiano, M., Garbarino, E., Lumachi, S., Solari, S., & Sanguineti, V. (2015, August). *Effect of interface type in the VR-based acquisition of pedestrian skills in persons with ASD*. Paper presented at the 37th Annual International Conference of the IEEE Engineering in Medicine and Biology Society (EMBC), Milan, Italy. 10.1109/EMBC.2015.7319693 10.1109/EMBC.2015.7319693 26737593

[CR35] Steam Client [Software] (2018). Bellevue.

[CR36] Strickland DC, McAllister D, Coles CD, Osborne S (2007). An evolution of virtual reality training designs for children with autism and fetal alcohol spectrum disorders. Topics in Language Disorders.

[CR37] Summers J, Tarbox J, Findel-Pyles RS, Wilke AE, Bergstrom R, Williams WL (2011). Teaching two household safety skills to children with autism. Research in Autism Spectrum Disorders.

